# Abnormal Ca^2+^ Spark/STOC Coupling in Cerebral Artery Smooth Muscle Cells of Obese Type 2 Diabetic Mice

**DOI:** 10.1371/journal.pone.0053321

**Published:** 2013-01-03

**Authors:** Angélica Rueda, María Fernández-Velasco, Jean-Pierre Benitah, Ana María Gómez

**Affiliations:** 1 Departamento de Bioquímica, Centro de Investigación y de Estudios Avanzados del IPN, México City, México; 2 Inserm, U-637; Université de Montpellier 1, Université de Montpellier 2, Montpellier, France; 3 Instituto de Investigación Hospital Universitario La Paz (IdiPAZ), Madrid, Spain; 4 Inserm, U769; Université de Paris-Sud, IFR141, Labex Lermit, Châtenay-Malabry, France; University of Debrecen, Hungary

## Abstract

Diabetes is a major risk factor for stroke. However, the molecular mechanisms involved in cerebral artery dysfunction found in the diabetic patients are not completely elucidated. In cerebral artery smooth muscle cells (CASMCs), spontaneous and local increases of intracellular Ca2+ due to the opening of ryanodine receptors (Ca2+ sparks) activate large conductance Ca2+-activated K+ (BK) channels that generate spontaneous transient outward currents (STOCs). STOCs have a key participation in the control of vascular myogenic tone and blood pressure. Our goal was to investigate whether alterations in Ca^2+^ spark and STOC activities, measured by confocal microscopy and patch-clamp technique, respectively, occur in isolated CASMCs of an experimental model of type-2 diabetes (*db/db* mouse). We found that mean Ca^2+^ spark amplitude, duration, size and rate-of-rise were significantly smaller in Fluo-3 loaded *db/db* compared to control CASMCs, with a subsequent decrease in the total amount of Ca^2+^ released through Ca^2+^ sparks in *db/db* CASMCs, though Ca^2+^ spark frequency remained. Interestingly, the frequency of large-amplitude Ca^2+^ sparks was also significantly reduced in *db/db* cells. In addition, the frequency and amplitude of STOCs were markedly reduced at all voltages tested (from −50 to 0 mV) in *db/db* CASMCs. The latter correlates with decreased BK channel β1/α subunit ratio found in *db/db* vascular tissues. Taken together, Ca^2+^ spark alterations lead to inappropriate BK channels activation in CASMCs of *db/db* mice and this condition is aggravated by the decrease in the BK β1 subunit/α subunit ratio which underlies the significant reduction of Ca^2+^ spark/STOC coupling in CASMCs of diabetic animals.

## Introduction

More than 65% of patients with diabetes die from cardiovascular disease or stroke [1]. When considering age-adjusted incidence rates, type-2 diabetic patients are two- to five times as likely to suffer cerebral vascular disease or stroke compared with non-diabetic patients, a disparity that is seen in multiple racial/geographic groups [2]–[6] and may result from abnormal cerebral artery tissue function. Interestingly, the incidence of stroke in type-2 diabetic patients is not associated with the duration of disease, smoking, fasting blood glucose, total cholesterol, lipoprotein concentrations, or hypertension [3], [4], [7]. Cerebral blood flow disturbances, impaired cerebral vascular reactivity, transient ischemic attacks and oxidative damage of cerebral vessels have been found in both type-2 diabetic patients [3] and experimental models [8]–[10] that could account for the higher incidence of diabetes-related stroke events [1]–[7]; however, the molecular mechanisms involved in cerebral artery dysfunction are not completely elucidated.

The *db/db* mouse, a genetic model of non-insulin dependent type-2 diabetes exhibits cerebral vascular dysfunction [9] that exacerbates brain damage, edema and inflammation after induced experimental stroke [11]–[13]. In addition to diabetes-related alterations found in cerebral vessels, vascular dysfunction is also present in mesenteric arteries [14]–[16], coronary arterioles [17], *gracilis* muscle arterioles [18], [19], and aorta [20]–[22] of *db/db* mice. Cerebral arterioles of *db/db* mice show impaired response to vasodilators and reduced baseline arteriolar diameter [9]. Mesenteric arteries and *gracilis* muscle arterioles of *db/db* mice show impaired response to vasodilators, enhanced response to vasoconstrictors and enhanced basal myogenic tone [14]–[16], [18], [23]. Consistent with the observations of augmented vascular tone, the myogenic pressure-diameter of arteries harvested from *db/db* mice are smaller than the diameters of corresponding control arteries [14], [18], [19] and are not improved by the removal of endothelium [14], [19]. Moreover, investigators have demonstrated impairment of endothelium-independent dilation in the presence of nitric oxide donors: in coronary arterioles and aorta of *db/db* mice in the presence of sodium nitroprusside [17], [20], and in arteries of type 2 diabetic patients after administration of glycerin trinitrate [24], [25]. All these data suggest that smooth muscle-dependent mechanisms are also responsible for the vascular dysfunction associated with type-2 diabetes. Furthermore, the disease appears to alter functional responses of resistance arteries not only at endothelial level but also in active smooth muscle layers.

In cerebral artery smooth muscle cells (CASMCs), spontaneous and local increases of intracellular Ca^2+^ due to the opening of Ryanodine Receptors (RyRs), visualized as Ca^2+^ sparks, activate large conductance Ca^2+^ sensitive K^+^ channels (BK channels) that generate spontaneous transient outward currents (STOCs) [26], [27]. STOCs have a key role in the control of arterial tone by shifting the membrane potential towards less positive values, which in turn limits Ca^2+^ influx through L-type Ca^2+^ channels, diminishes global intracellular Ca^2+^ concentration ([Ca^2+^]_i_), and opposes vasoconstriction [28]–[30]. Therefore, RyRs through Ca^2+^ sparks and BK channels, by producing STOCs regulates arterial tone favoring vasorelaxation [29]–[31]. In addition, Ca^2+^ spark generation is also regulated by Ca^2+^ influx due to an indirect coupling between L-type Ca^2+^ channels and RyRs [32]. The sarco/endoplasmic reticulum Ca^2+^ ATPase (SERCA) participates in this indirect coupling by redirecting the Ca^2+^ coming from the extracellular medium towards its luminal stores, located mainly inside the sarcoplasmic reticulum (SR) of CASMCs. Navedo and collaborators [33] demonstrated that L-type Ca^2+^ currents and Ca^2+^sparklet activity –brief plasma membrane Ca^2+^ fluxes due to the activation of clusters of L-type Ca^2+^ channels– are enhanced in CASMCs of *db/db* mice; suggesting that the mechanisms that participate in the closure and tight regulation of L-type Ca^2+^ channels could be impaired in diabetic CASMCs. Therefore, we sought to investigate whether BK channels and their functional pair, the RyRs, might contribute to worsening the condition of diabetic CASMCs.

Our data represent the first demonstration of altered Ca^2+^ spark and STOC in CASMCs from *db/db* mice due to Ca^2+^ spark alterations which lead to inappropriate BK channels activation, and the latter is aggravated by the decrease in the BK β1 subunit/α subunit ratio also found in CASMCs of *db/db* mice.

## Results

### Reduced Spatiotemporal Properties of Ca^2+^ Spark in CASMCs of Diabetic Mice

Ca^2+^ sparks of CASMCs represent the spontaneous and coordinated opening of an undefined number of RyRs within a cluster [26], [30]. Their frequency and properties –*i.e.* amplitude, duration, size and mean rise rate– are used as markers of *in situ* RyR activity [34]. Since impaired function of RyRs might contribute to the vascular alterations in the cerebral arteries of diabetic *db/db* mice [8], [9], [33], our first aim was to study the *in situ* activity of vascular RyR in single, freshly isolated CASMCs of diabetic mice.

**Figure 1:**
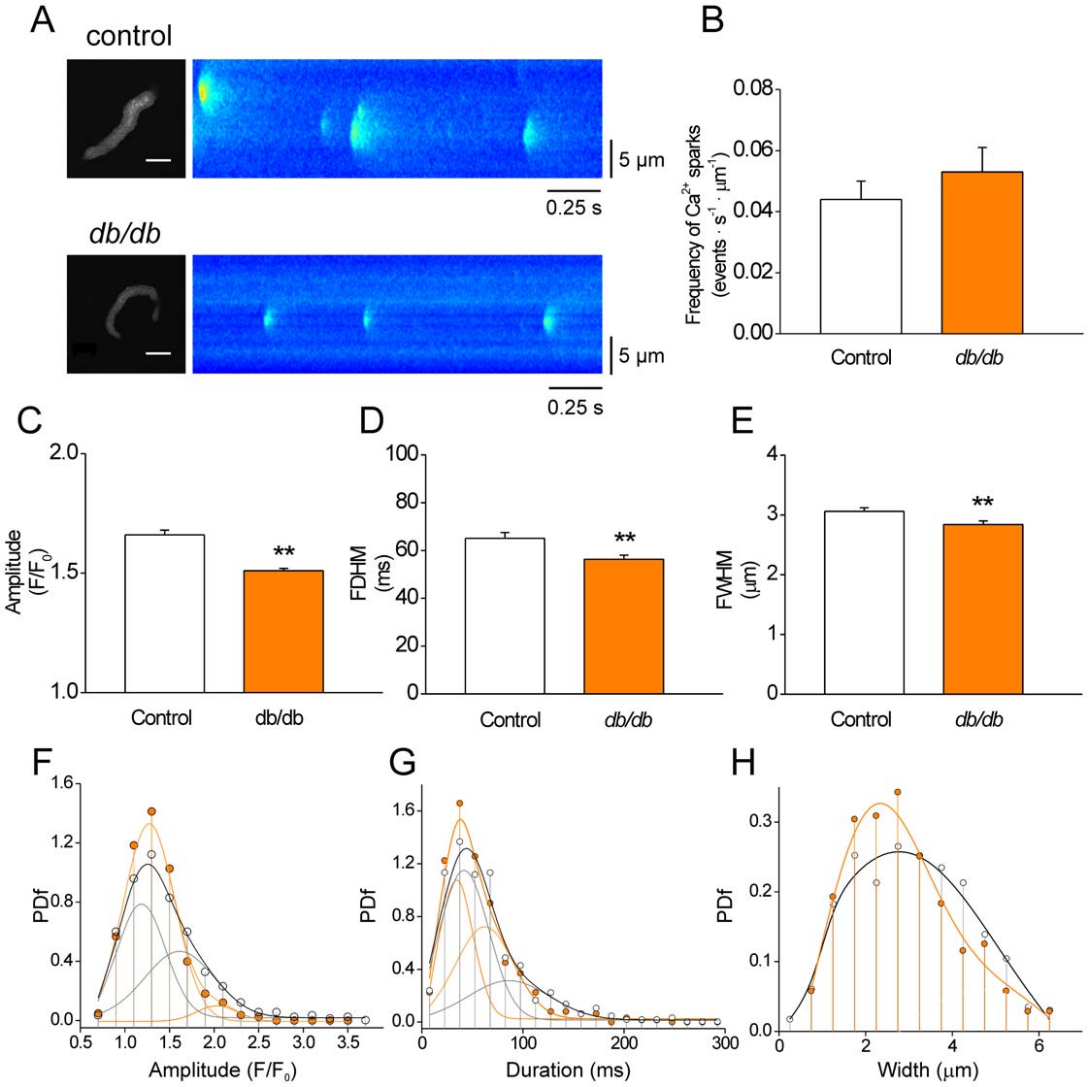
Reduced properties of spontaneous Ca^2+^ sparks in CASMCs of diabetic mice. A. Confocal images of Fluo-3 loaded CASMCs (*left*) and normalized representative line scan images (1.92ms/line, *right*) showing spontaneous Ca^2+^ sparks recorded in control (*top images*) and *db/db* (*db/db*; *bottom images*) cells. Intact CASMCs are relaxed and show their typical spindle shape. Scale bar = 15 µm. **B**. Bar graph of Ca^2+^ spark frequency –measured as number of Ca^2+^events observed per second per µm– in 43 intact control (*white bars*) and 41 *db/db* (*orange bars*) CASMCs**. C-E** Bar graphs of averaged Ca^2+^ spark amplitude (**C**, F/F_0_); duration (**D**, FDHM or full duration at half maximum in ms); and width (**E**, FWHM, full width at half maximum in µm). **F-H**. Probability density function (PDf) of Ca^2+^ spark amplitudes (**F**; F/F_0_), durations (**G**; ms), and widths (**H**; µm) in control (*white circles*; n = 460) and *db/db* (*orange circles*; n = 423 events) CASMCs. Curves represent mixed gaussian functions fitted to the data.

Figure 1*A* shows representative confocal images of Ca^2+^sparks (*right images*) recorded in intact control and diabetic (*db/db*) CASMCs that conserve their typical relaxed spindle shape (*left images*). We found that the *db/db* CASMCs produced similar Ca^2+^ spark frequency to control cells (in Hz: 0.8±0.1 *vs.* 0.8±0.1; or in events·s^−1^·µm^−1^: 0.053±0.008 *vs.* 0.044±0.006, in 41 *db/db* cells and 43 control cells, respectively (Fig.1*B*), with no further modifications in the number of firing sites per cell, eager site probability and the maximum number of events within a same site (Figure S1). The maintained Ca^2+^ spark frequency in CASMCs contrasts with the decrease found in cardiac myocytes of the same mouse model [35]. However, *db/db* CASMCs presented a significant reduction in average Ca^2+^ spark amplitude (Fig. 1C), full duration at half maximum (FDHM, Fig. 1D), full width at half maximum (FWHM, Fig. 1E) and mean rising rate (d(F/F_0_)/dt: 0.181±0.006 *vs.* 0.163±0.005 in 460 Ca^2+^ sparks from 43 control CASMCs *vs.* 423 Ca^2+^ sparks from 41 *db/db* CASMCs, *P*<0.05).

**Figure 2:**
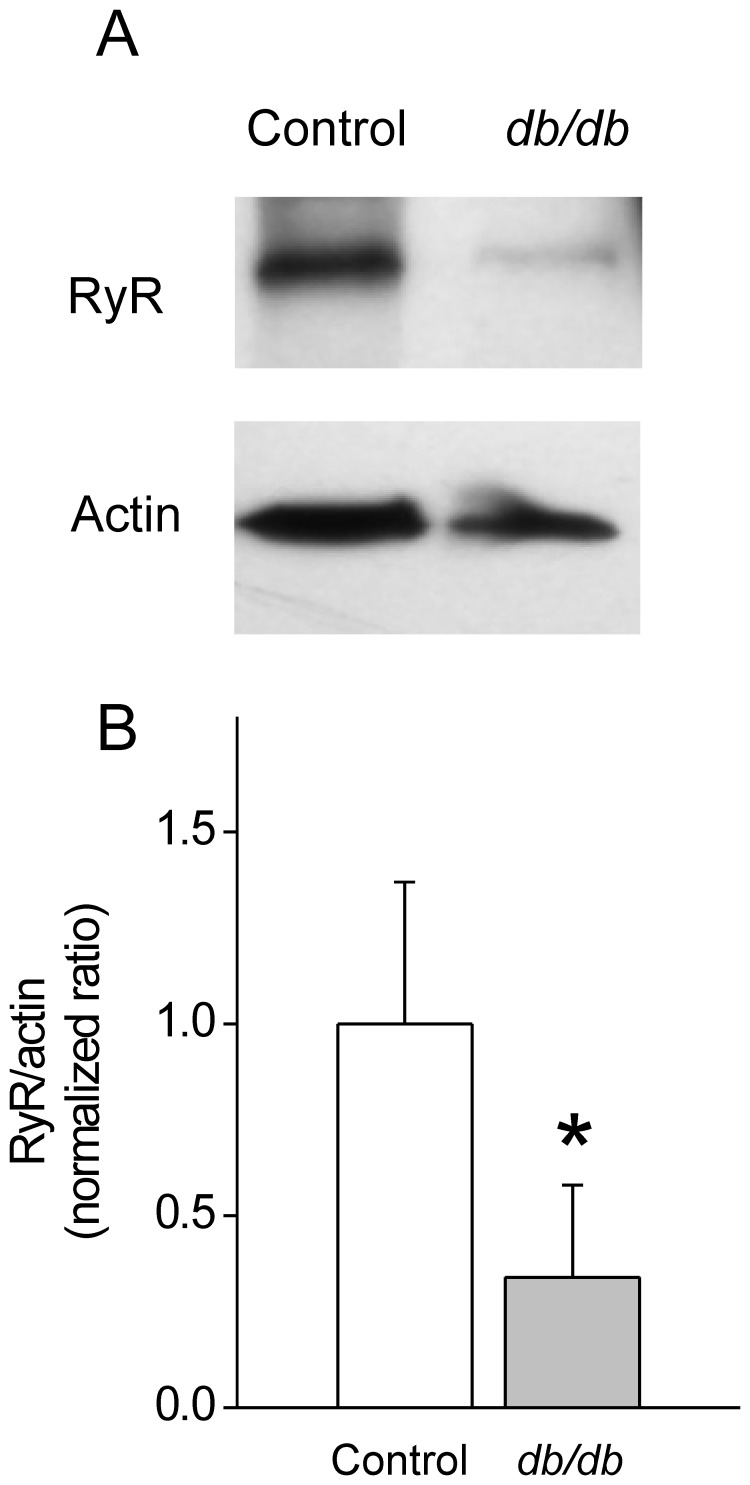
Decreased Ryanodine Receptor expression in vascular tissue homogenates of *db/db* mice. A . Representative Western Blots of RyR obtained from control and *db/db* vascular tissue homogenates. Samples containing 30 µg of protein were run on 15% acrylamide gels and probed with anti-RyR antibody (1∶5000) and anti-actin (1∶8000). **B**. Bar graph represents normalized RyR/actin densitometric ratios (n = 3 for each group).

Similar to previous observations in cardiac cells [35], histogram distributions of Ca^2+^ spark parameters –plotted as a function of probability density (PDf)– were asymmetrical for both control and diabetic events (Fig 1*F–H*) and were fitted to a bimodal Gaussian function [35]. The amplitude distributions (Fig 1*F*) showed 2 peaks: one around 1.25 F/F_0_ and another of around 1.6 F/F_0_; however, the population of events at larger amplitudes was drastically reduced in *db/db* compared to control CASMCs (43.7% in control cells to 5.9% in *db/db* cells, Table 1), which accounted for the decrease in the average Ca^2+^ spark amplitude. In addition, the distribution of Ca^2+^ spark durations showed 2 peaks that in *db/db* cells were shifted towards shorter durations (in ms: first peak from 41.80 to 34.11 and second peak from 86.16 to 62.37, Table 1) with modified proportions (in %: first peak from 69.3 to 43.9 and second peak from 30.0 to 49.6). On average, these effects decreased the population of longer Ca^2+^ sparks in *db/db* CASMCs (Figure1*G*). In the case of size (FWHM) distribution (Fig 1*H*) the population of events changed from one peak distribution (mean at 2.77 µm) in controls to two peaks distribution (in µm: first peak at 2.03, second peak at 4.69, Table 1). Taken together, these results show a shift of the population to weaker, shorter and smaller Ca^2+^ sparks in *db/db* CASMCs. Since Ca^2+^ spark amplitude, duration and mean rising rate are good indicators of local release flux [34], we can conclude that although the total occurrence of Ca^2+^ sparks are similar between *db/db* and control CASMCs, the total Ca^2+^ mass (calculated by amplitude*duration*width) released through Ca^2+^ sparks was decreased in *db/db* cells (in F/F_0_·ms·µm: 317.0±18.2 in 423 *db/db* events *vs.* 472.1±30.8 in 460 control events, *P*<0.001). This could be due to a decreased number of RyR opening simultaneously in a cluster. We estimated RyR expression by western blots in aorta homogenates and found a decrease in *db/db* mice (Figure 2).

**Table 1:** Mean (µ), SE(σ), proportion (*p* in %) and correlation coefficient (*r* ^2^) of Gaussian Fits to Ca^2+^ spark parameters.

Amplitude (F/F_0_)							
	µ_1_	SE_1_	*p*_1_	µ_2_	SE_2_	*p*_2_	*r^2^*
control	1.19	0.08	51.8	1.61	0.77	43.7	0.99
*db/db*	1.28	0.01	94.1	2.03	0.08	5.9	0.99
**Duration (ms)**							
	**µ_1_**	**SE_1_**	***p*** **_1_**	**µ_2_**	**SE_2_**	***p*** **_2_**	***r^2^***
control	41.80	5.27	69.3	86.16	162.0	30.0	0.95
*db/db*	34.11	1.32	43.9	62.37	21.18	49.6	0.99
**Width (µm)**							
	**µ_1_**	**SE_1_**	***p*** **_1_**	**µ_2_**	**SE_2_**	***p*** **_2_**	***r^2^***
control	2.77	0.22	100	0.0	0.0	0.0	0.97
*db/db*	2.03	3.36	86.2	4.69	32.8	13.8	0.98

### Sarcoplasmic Reticulum Ca^2+^ Loading CASMCs from Diabetic Animals

**Figure 3:**
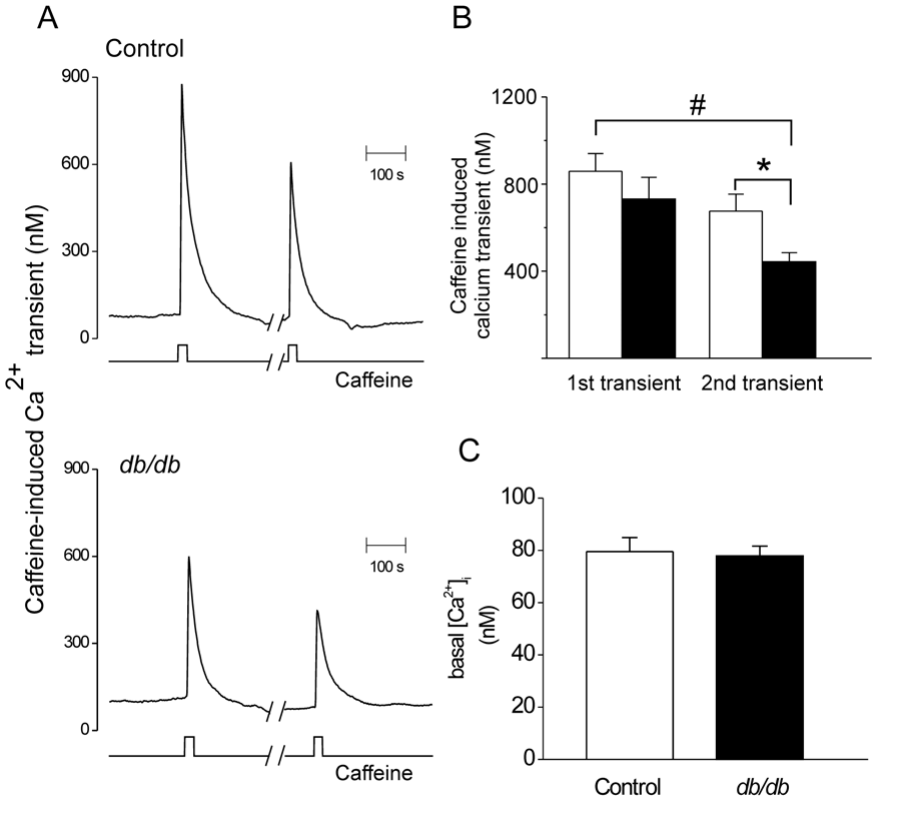
SR Ca^2+^ load in CASMCs of diabetic mouse. A . Representative caffeine-induced Ca^2+^ transients in Fura-2 loaded CASMCs from control (*upper graph*) and *db/db* mice (*bottom graph*); bottom traces indicate the start and end of caffeine applications (10 mmol/L). Breaks in x-axis indicate a 5-min interval between the first and the second caffeine application to allow the recovery of SR Ca^2+^ stores. **B**. Bar graph of averaged caffeine-induced Ca^2+^ transients. *P<0.05 **C**. Bar graph of averaged basal [Ca^2+^]_i_ concentrations in CASMCs from control *(open bar*, n = 50 cells) and *db/db* mice (*fill bar*, n = 62 cells).

Sarcoplasmic reticulum (SR) Ca^2+^ load is a major determinant of Ca^2+^ release regulating RyR opening and, by consequence, Ca^2+^ spark properties and activity. It is plausible, therefore, that a reduced SR Ca^2+^ load could contribute to the reduction in Ca^2+^ spark properties described from CASMCs of diabetic mice. Thus, we measured global SR Ca^2+^ content by rapid application of caffeine (10 mmol/L). Global [Ca^2+^]_i_ responses were measured in CASMCs from control and *db/db* mice using fura-2 based Ca^2+^ microfluorometry. Examples of such traces are shown in Fig. 3*A*, *left traces.* No significant differences in the peak amplitude of the caffeine-evoked [Ca^2+^]_i_ transients were observed (in nmol/L: 858.6±81.6, n = 28 control cells *vs.* 731.4±99.5, n = 18 *db/db* cells) (Fig. 3*B*, *left bars*) thus ruling out a difference on the SR Ca^2+^ load at rest between *db/db* and control CASMCs underlying the weaker Ca^2+^ sparks. To observe the time dependence of luminal Ca^2+^ replenishment [36], we allowed 5 min of recovery and applied caffeine again. In this case, the peak amplitude of the second [Ca^2+^]_i_ transient was significantly reduced in the CASMCs of diabetic animals (in nmol/L: 675.9±78.2, n = 16 control cells *vs.* 443.1±41.9, n = 13 db/db cells, *P*<0.05) (Fig. 3*B*). The normalized SR Ca^2+^ load recovery with respect to the first caffeine-induced [Ca^+^]_i_ transient was of 62.6±0.08% for db/db CASMCs, *vs.* 78.4±0.1% for control CASMCs. Thus the time dependence of SR Ca^2+^ load recovery is impaired in the vascular cells of diabetic mice, with no alterations in basal [Ca^2+^] (74.5±5.1 nmol/L, n = 23 control *vs.* 76.4±6.9 nmol/L, n = 21 db/db CASMCs. Fig. 3*C*), suggesting that SERCA pump function might be compromised.

### Decreased STOC Activity in CASMCs from Diabetic Mice

**Figure 4:**
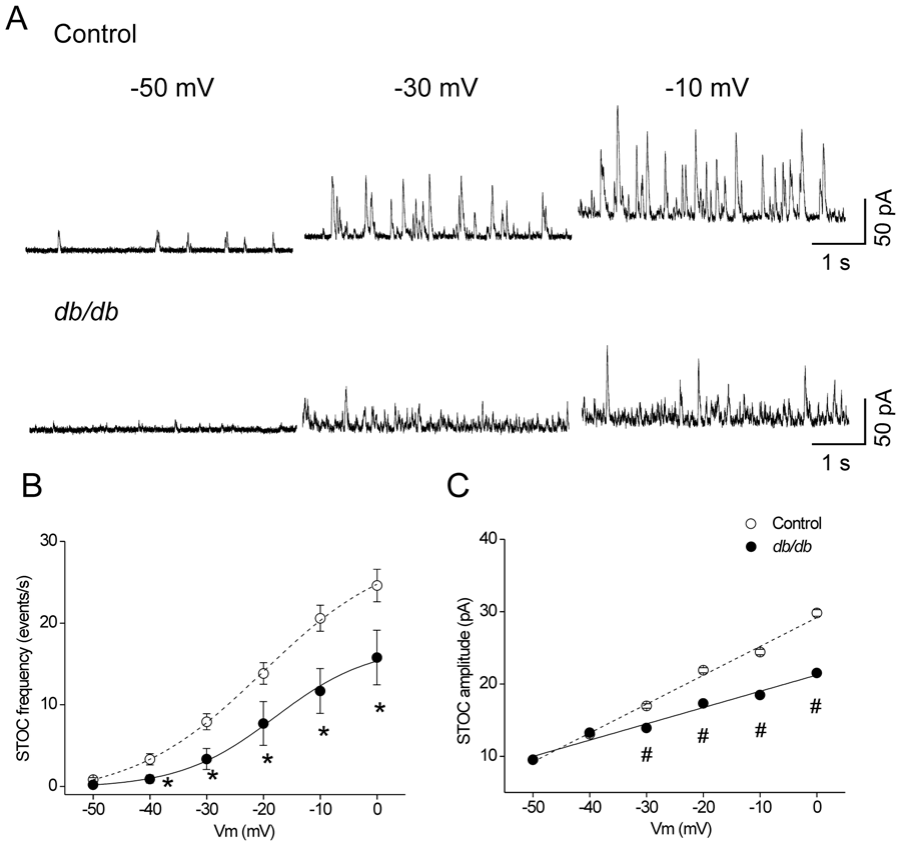
Decreased STOC frequency and amplitude in CASMCs of *db/db* mice. A . Representative traces of STOC activity at different holding potentials (−50, −30 and 0 mV) obtained from freshly isolated control (*upper traces*) and *db/db* (*bottom traces*) CASMCs. Voltage dependence of STOC frequency (**B**) and amplitude (**C**); cells were voltage clamped at different holding potentials (from −50 to 0 mV) and the STOC activity was recorded. The plots show STOC frequencies and amplitudes (M ± SEM) from 9 controls (*open circles, dash line*) and 8 *db/db* (*filled circles, solid line*) CASMCs at each holding potential. *P<0.05 and #*P*<0.01; control vs. *db/db* cells.

**Figure 5:**
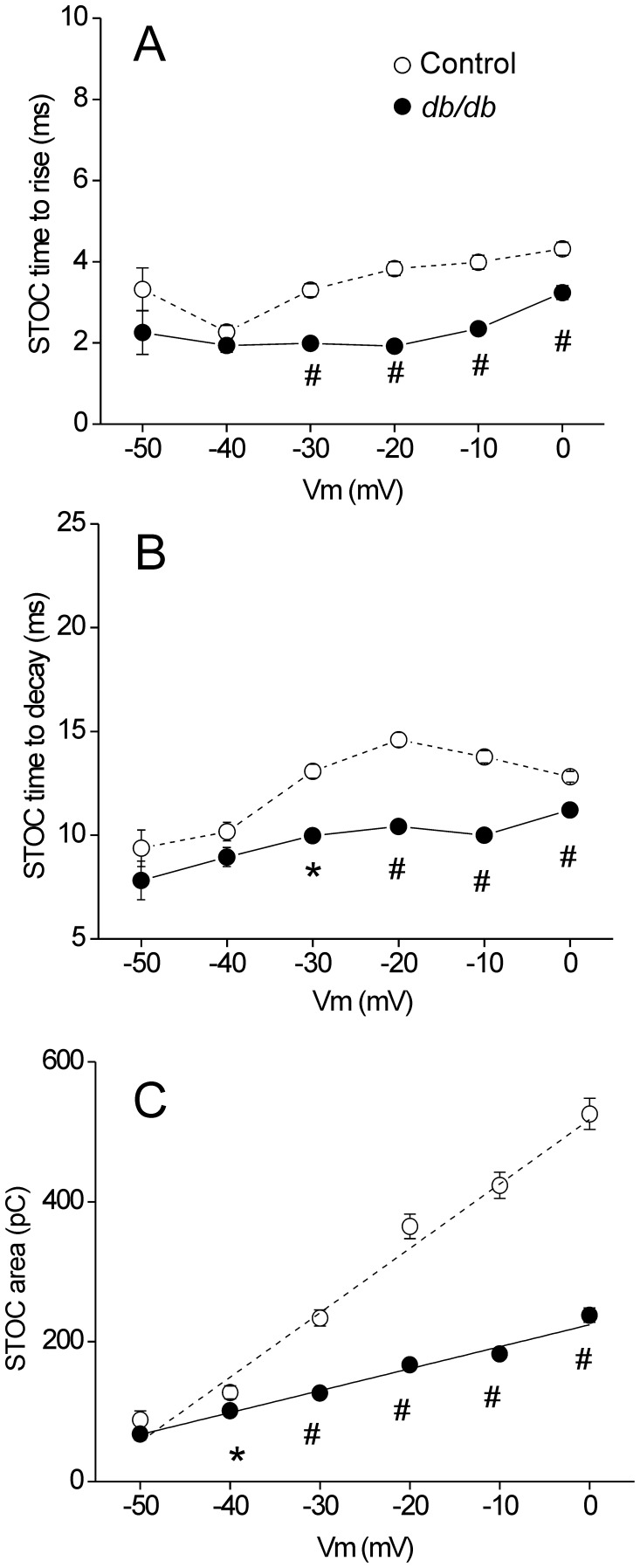
Reduced properties of STOCs from diabetic CASMCs. Voltage dependence of STOC time-to-rise (**A**), time-to-decay (**B**) and area (**C**). Cells were voltage clamped at different holding potentials (from −50 to 0 mV) and the STOC activity was recorded. The plots show STOC properties (M ± SEM) of 9 controls (*open circles, dash lines*) and 8 diabetic (*filled circles, solid lines*) CASMCs at each holding potential. *P<0.05 and #*P*<0.01; control *vs.* diabetic cells.

In CASMCs, Ca^2+^sparks activate large conductance K^+^ (BK) channels, which play a critical role in controlling vascular tone [26], [30]. We aimed to investigate whether the smaller Ca^2+^ sparks in diabetic CASMCs had an impact on BK channel activity by analyzing spontaneous transient outward currents (STOCs). Intact CASMCs were patch-clamped and progressively depolarized from a holding potential of −50 mV to 0 mV in 10 mV voltage increments (Fig. 4*A*). STOC frequency was significantly decreased in db/db CASMCs compared to control cells across a range of voltages from −40 mV to 0 mV (Fig. 4*B*). However, sigmoidal fitting of the data revealed that the voltage dependence of STOC frequency was unaffected (voltage of half maximum activation, V_50_: −20.1±0.9, and −17.9±2.9 mV, in 9 control and 8 *db/db* CASMCs, respectively), suggesting that voltage sensitivity of BK channel activation is unaltered in type 2 diabetes. Amplitude was also significantly reduced in db/db CASMCs as compared to control cells, with statistical difference from −30 mV to 0 mV. However, linear fitting of these data demonstrated that the voltage dependence of STOC amplitude was diminished 0.55 fold in *db/db* CASMCs (slope: 0.40±0.01, vs. 0.22±0.02 pA/mV; Fig. 4*C*). The diminished STOC amplitude in the diabetic CASMCs was not due to a change in cell size, since there was no significant difference in membrane capacitance (an indicator of cell surface) between diabetic and non-diabetic CASMCs (9.5±2.4 pF for *db/db* n = 10 cells *vs.* 10.0±1.5 pF for control n = 15 cells) nor to a change in zero current potential (−33.5±2.1 mV in 7 WT cells, *vs.* −33.1±3.4 mV in 5 *db/db* cells). Further analysis of STOC kinetics revealed that both STOC time-to-rise (Fig. 5*A*) and time-to-decay (Fig. 5*B*) had shorter durations in *db/db* CASMCs compared to control. Furthermore, STOC area was significantly diminished in *db/db* CASMCs *vs* control cells (Fig. 5*C*).

### The Proportion of β1 to α Subunit Expression of BK Channels is Decreased in *db/db* CASMCs

**Figure 6:**
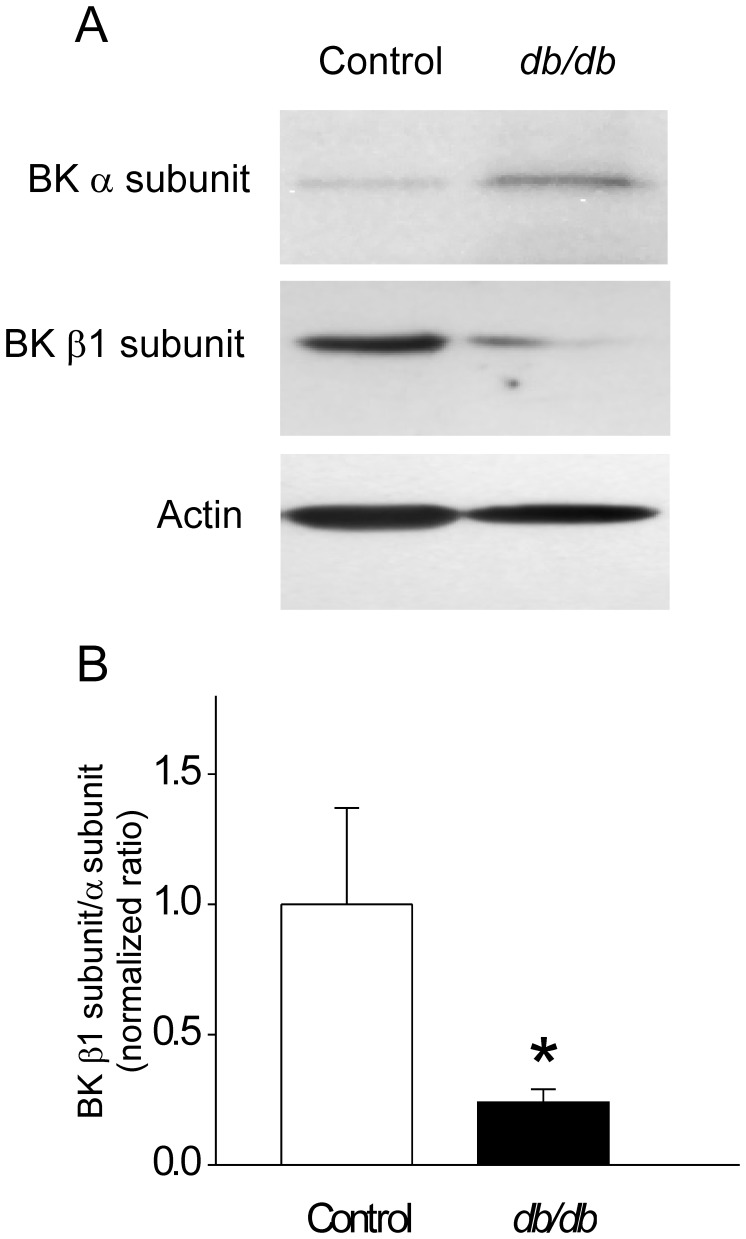
Decreased BK β1/α -subunit ratio in vascular tissues of *db/db* mice. **A**. Representative Western Blots of BK α and β1 subunits obtained from control and *db/db* vascular tissues. Samples containing 30 µg of protein from aorta whole homogenates were run on 15% acrylamide gels and probed with anti-BK α subunit antibody (1∶1000), anti-BK β1 subunit antibody (1∶500) and anti-actin (1∶8000). **B**. Bar graph represents normalized BKβ1/α densitometric ratios (n = 7 for each group). *P<0.05.

We next sought to determine whether the diminished frequency and amplitude of STOCs in the diabetic CASMCs might be related to a change in BK channel composition. Vascular BK channels are composed of four pore-forming α-subunits and four accessory β-subunits, β1 being the predominant isoform expressed in vascular smooth muscle [37]. The α-subunit is the pore forming subunit, and the β1-subunit enhances the sensitivity of BK channels to Ca^2+^-dependent activation [37]. Downregulation or genetic disruption of β1 subunit has been implicated in the pathogenesis of diabetic vascular dysfunction [38]–[41] and arterial hypertension [42]. We determined protein expression of BK channel α- and β1-subunits in aorta homogenates of control and *db/db* mice. Anti-actin antibody was used to normalize the loading. Representative immunoblots (Fig. 6*A*) and bar graphs (Fig. 6*B*) show that BK β1/α subunit ratio is 4.2-fold decreased in the diabetic mice (normalized ratio to actin: 1.0±0.37 *vs.* 0.24±0.05, in n = 7 controls and n = 7 *db/db* tissue homogenates, *P*<0.05); suggesting that composition stoichiometry of BK channels is modified by the diabetic condition; with decreased proportion of BK channel β1-subunits to α-subunits. Separate analysis of BK α and β1 subunit expression levels were slightly but not significantly modified (BKα normalized ratio to actin: 1.0±0.3 in controls *vs.* 1.6±0.7 in diabetic samples; BKβ1 normalized ratio to actin: 1.0±0.3 in n = 7 controls *vs.* 0.6±0.2 in n = 7 diabetic samples).

### Reduced STOC/spark Coupling in CASMCs of Diabetic Mice

**Figure 7:**
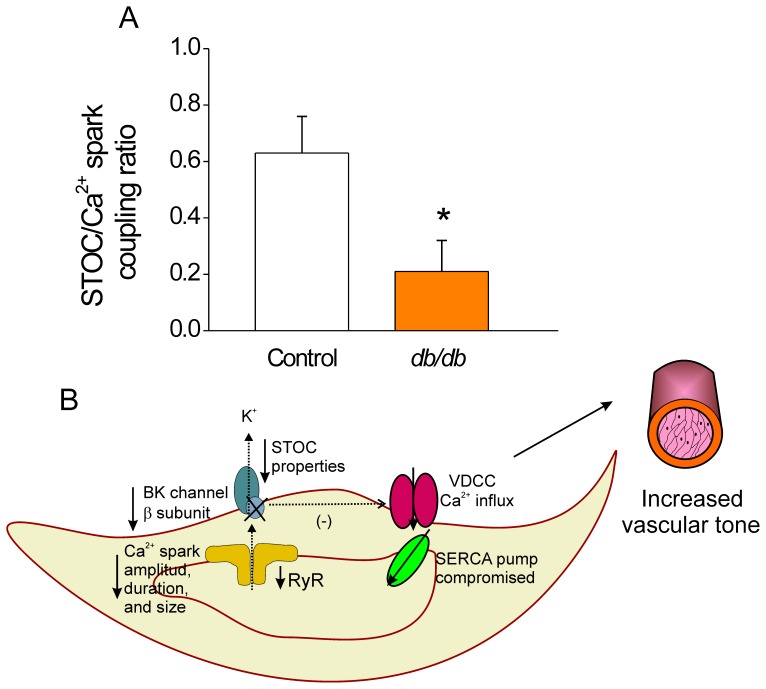
Reduced STOC/spark coupling as a factor contributing to an increased vascular tone in diabetic CASMCs. A . Estimated STOC/Spark coupling ratio in control (*white bar*) and *db/db* (*orange bar*) CASMCs. The estimated coupling ratio was obtained by dividing the frequency of STOCs in each cell (n = 11 control *vs* n = 10 *db/db* cells) at near resting membrane potential (−40 mV) between the average frequency of spontaneous Ca^2+^ sparks in single quiescent cells; *P<0.05. **B**. Schematic representation of single *db/db* CASMC where the triggering of STOCs is not tightly controlled by Ca^2+^ sparks. The decreased STOC/Spark coupling ratio suggests that most of the Ca^2+^ sparks produced in the diabetic cells are STOCless having a detrimental impact on the regulation of vascular myogenic tone. SERCA: sarco/endoplasmic reticulum Ca^2+^ ATPase, BK: big conductance Ca^2+^-activated K^+^ Channel, VDCC: L-type Ca^2+^ channel, RyR: ryanodine receptor.

It has been shown that Ca^2+^ sparks have a vasorelaxing effect by activating STOCs, which hyperpolarizes the cell, leading to inactivation of VDCCs. The STOC/Ca^2+^spark coupling ratio was indirectly estimated by dividing the number of STOCs at near resting membrane potential (−40 mV) by the number of spontaneous Ca^2+^ sparks in single quiescent cells. Fig. 7A shows that this coupling ratio was decreased in *db/db* CASMCs, suggesting that many of the Ca^2+^ sparks produced in the diabetic CASMCs lack STOCs, contrary to what is observed in normal vascular myocytes [29]. The estimated reduced coupling ratio may have a considerable impact on the deregulation of vascular tone under the diabetic condition.

## Discussion

In this work we present the first compelling evidence of the RyR and BK channel functional loss in native CASMCs of *db/db* mice. We have demonstrated: 1) Ca^2+^ spark properties are significantly decreased in the CASMCs of diabetic animals; 2) STOC frequency, amplitude, kinetics and area were further reduced; and 3) diminution of the BK channel β1/α subunit ratio, resulting in an abnormal Ca^2+^ spark-STOC coupling in *db/db* CASMCs.

Taken together, our data suggests the scenario schematized in Fig. 7B where the triggering of STOCs is not tightly controlled by Ca^2+^ sparks in db/db CASMCs, probably due to 1) smaller and shorter Ca^2+^ sparks with reduced amplitude than then could be STOCless; 2) a compromised SR Ca^2+^ load recovery; and 3) decreased STOC frequency, amplitude, area, time-to-rise and time-to-decay. The latter is probably due to reduced Ca^2+^ sparks because of decreased RyRs expression and further enhanced by the altered subunit composition of BK channels. Taken together, the reduced efficacy of Ca^2+^ sparks to trigger STOCs might contribute to an increase in vascular tone of *db/db* arteries.

Previous studies have shown that the function of cerebral arteries is impaired in experimental and genetic models of type-2 diabetes [8]–[10], [41]. Specifically, the cerebral vascular dysfunction found in the *db/db* mice is characterized by reduced baseline arteriolar diameter, increased basal tone and reduced response to acetylcholine, in part due to enhanced Rho-kinase activity, superoxide production [9] and overall oxidative damage [8] that worsens brain damage, edema and inflammation after induced experimental stroke [11]–[13]. In the current study, we have analyzed intracellular Ca^2+^ handling and STOCs which might contribute to these alterations, since it has been previously shown that spontaneous Ca^2+^ sparks contribute to vascular relaxation by activating STOCs.

We found that the overall Ca^2+^ spark frequency was not significantly altered, but the Ca^2+^ spark amplitude, duration, width, and as a consequence Ca^2+^ spark mass were decreased in *db/db* CASMCs. To our knowledge, this is the first study of Ca^2+^ sparks in CASMCs of type 2 diabetes. In type 1 diabetes, Ca^2+^ sparks frequency has been found to be unaltered [40] or increased [39], while their amplitudes were found to be increased [39], [40]. Although both type 1 and type-2 diabetes are characterized by hyperglycemia, the mechanism of disease is quite different, which may explain the differing results in Ca^2+^ spark amplitude.

It has been previously established that decreasing SR Ca^2+^ load in vascular myocytes reduces Ca^2+^ spark properties [43]. We did not find a significant decrease in the SR Ca^2+^ load in the conditions of Ca^2+^ sparks recording (Fig. 3), ruling out this possibility. However, the recovery of SR Ca^2+^ load after 5 min of depletion was impaired in *db/db* mice, which could be indicative of an underlying defect in SERCA activity. In fact, it has been documented that cytokines (i.e. interleukin-1 βand γ-interferon), which are increased in the *db/db* mice, depress SERCA function [44]. On the other hand, reduced number of RyRs that simultaneously open in a cluster may account for the reduction in Ca^2+^ spark amplitude, duration, spatial spread, and mass of the Ca^2+^ spark. This could be supported by the reduced RyR expression in db/db arteries (Fig. 2).

Ca^2+^ sparks in vascular smooth muscle cells contribute to relaxation by activating BK channels. Diabetic CASMCs show a significant decrease in STOC frequency, amplitude, time-to-rise, time-to-decay and area (Fig. 4 and 5), as in type 1 diabetes [39], [40]. STOCs are outward (hyperpolarizing) currents mediated by BK channels. The K^+^ efflux brings the cell membrane to more negative potentials, limiting the activation of voltage-dependent Ca^2+^ channels. This in turn decreases Ca^2+^ influx, inducing relaxation [26]–[31]. Hence, the decrease in Ca^2+^ sparks properties, STOCs and Ca^2+^ sparks-STOCs coupling could underlie or contribute to depolarization under certain conditions *in vivo*.

Because STOCs are activated by Ca^2+^ sparks, and the occurrence of Ca^2+^ sparks is not significantly altered, the reduction in STOCs could be interpreted as a decreased ability of the Ca^2+^ sparks to activate STOCs in *db/db* CASMCs (Fig. 7A). There are multiple potential explanations for this uncoupling. It is possible that the smaller Ca^2+^ sparks are less effective in activating STOCs. Indeed, after careful analysis of Ca^2+^ spark amplitude, duration, and width PDf (Fig. 1F–H), we found that the global decrease in Ca^2+^ spark properties was related to a decrease in the population of larger Ca^2+^ sparks. Additionally, the reduction in STOCs could be due to the relative decrease in β1 channel subunit (Fig. 6) [38]–[41], [45], since it has been shown that the β-subunit plays a major functional role in defining both the Ca^2+^ dependence of activation and availability of BK channels [37]. In this regard, BK channels of coronary arteries from Zucker diabetic fatty rats showed impaired Ca^2+^ dependent activation attributable to a decrease in BK channel β1-subunit expression [38]. We found a decrease in the β1/α BK subunit proportion (Fig. 7), suggesting that in *db/db* mice, some BK channels may be devoid of β1-subunit. Additional biochemical studies of type-1 diabetic animals also support the evidence that BK channel β1-subunit relative to BK channel α-subunit protein expression is reduced in arterial tissues [39], [40]. Interestingly, similar findings have been reported in CASMCs of spontaneously hypertensive rats [42] underlying a common mechanism to decrease STOC/Ca^2+^spark functional coupling. Additionally, a key finding of this study is that the function of BK channels is impaired in CASMCs of type-2 diabetic mice, but that this is not attributable to an altered voltage dependence of BK channel activation. Although we cannot exclude the possibility that the dysfunction of BK channels found in diabetic CASMCs could be due to high glucose-mediated oxidative modulation [46], we suggest that Ca^2+^ sparks with diminished amplitude, size, duration, and mass may be less efficient in activating STOCs. Indeed, the STOC/Ca^2+^ spark coupling efficiency was significantly reduced in *db/db* cells (Fig. 7A), which suggests that spark/STOC coupling plays a key role in contributing to the previously well documented vascular dysfunction associated with this model of type 2 diabetes [9]. Further, the decreased STOC activity in *db/db* mice could underlie the increased activity of L-type Ca^2+^ channels reported by Navedo *et al*. [33].

In summary, we have dissected local Ca^2+^ signaling-STOC activity in CASMCs from *db/db* mice and their controls. We have shown that the alteration in RyR activity, manifested as a decrease in the larger Ca^2+^ spark population, is involved in reduced STOC properties, with consequent dysfunction. Thus, defective crosstalk between RyRs and BK channels may be important contributors to the vascular pathology of diabetic individuals and, as such, are potentially attractive targets for therapeutic intervention.

## Materials and Methods

All experiments were carried out according to European Union Council Directives (86/609/EEC) for the care of laboratory animals. Animal Protocol was approved by the *Comité Régional d’Ethique sur l’expérimentation animale of Languedoc-Roussillon* on the Use and Care of Animals (authorization B34-172-16 for animal facility manager).

### Mice

The diabetic (*db/db*) mice used in the current study were on a C57BL/KsJ genetic background. We used male animals at the age of 14 to 16 weeks. *db/db* mice were significantly heavier than WT controls (body weight: 55.5±0.8 g for *db/db*, n = 17 animals *vs.* 30.0±0.8 g for controls, n = 13; *P*<0.001).

### CASM Cell Isolation

Cerebral artery smooth muscle cells (CASMCs) were enzymatically isolated from 14 to 16 week-old male C57BL/KsJ-db (*db/db*) mice and their controls (+/+), using a previously standardized method [47]. Mice were anesthetized by peritoneal injection of pentobarbital solution (100 mg/kg) plus heparin (4000 U/kg). The brain was removed and transferred to a Petri dish filled with oxygenated ice-cold HEPES-buffered dissection solution (HBDS, containing in mmol/L: 55 NaCl, 6 KCl, 80 Na-glutamate, 2 MgCl_2_, 10 glucose and 10 HEPES, pH 7.4). The cerebral basilar arteries were dissected, transferred to a 1.5 ml tube and incubated in the shaker for 16 min at 37 °C in HBDS containing in mg/ml: 1.0 papain (2X crystallized), 1.0 bovine serum albumin (BSA), and 1.0 dithiothreitol. Then, the tissue was transferred to a collagenase solution (mixture of collagenases type F and type H, 7∶3 ratio) plus 100 µmol/L CaCl_2_ and incubated for 8 min at 37 °C with shaking. Enzyme digestion was stopped by washing the tissue 5 times with HBDS. Digested tissue was triturated with a fire-polished glass Pasteur pipette to yield single smooth muscle cells. Myocytes used for confocal experiments were loaded with 10 µmol/L Fluo-3 AM for 30 min at room temperature, washed and kept at 4 °C in physiological saline solution (PSS, composition in mmol/L: 137 NaCl, 5.4 KCl, 1.8 CaCl_2_, 1 MgCl_2_, 10 glucose and 10 HEPES, pH 7.4) to be used within the same day.

### Ca^2+^spark Recordings

Spontaneous and local Ca^2+^ release events were recorded as previously reported [47]. Fluo-3 loaded CASMCs (50 µl of cell suspension) were allowed to adhere to the bottom of a glass coverslip in a perfusion chamber. The cells were perfused with physiological saline solution (PSS) at room temperature before starting the experiments. Ca^2+^ sparks were recorded at room temperature with a laser scanning confocal microscope (Zeiss, LSM510 META) equipped with an x63 water-immersion objective (N.A. 1.2) in the line scan mode (5 images of 1000 lines at speed of 1.92 ms/line). Fluo-3 was excited at 488 nm with an Argon laser (3% intensity), and emission measured at above 510 nm. Ca^2+^ sparks were reconstructed by stacking consecutive line scans and performing a time-intensity plot. Ca^2+^ sparks were automatically detected and properties of amplitude (F/F_0_), duration (FDHM, Full duration at half maximum, in ms) and width (FWHM, Full width at half maximum, in µm) measured with a custom-made program running in IDL 5.5 software (Research Systems Inc.), with a detection threshold of 4.3×S.D [36]. Images of Ca^2+^ sparks were normalized by dividing the fluorescence intensity of each pixel (F) by the average resting fluorescence intensity (F_0_) to generate an F/F_0_ image. Mean rate of rise was determined as reported by Shen *et al* [34].

### [Ca^2+^]_i_ Transients in CASMCs

Myocytes were loaded with 2.5 µmol/L fura 2-AM. Cells were transferred to a bath perfusion chamber and continuously superfused with PSS. Two caffeine-induced [Ca^2+^]_i_ transients were elicited consecutively with a 5-minute interval in between to allow refilling of intracellular Ca^2+^ stores. Images of Fura-2 fluorescence were captured by a cooled CCD camera (Photometrics, USA) with an oil-immersion x40 objective mounted on an inverted microscope (Axiovert, Zeiss, Germany), and acquired at 510 nm emission after excitations at 340 nm and 380 nm using a lambda-DG4 excitation system (Sutter Instrument Company, USA). Ca^2+^ signals were analyzed using Metafluor software (Universal Imaging Corporation, USA). [Ca^2+^]_i_ (in nmol/L) was calculated from the ratio of 340/380 nm after correcting for background. Fluorescence emission ratios were converted to [Ca^2+^]_i_ according to the Grynkiewicz equation [48], using a dissociation constant for fura-2 of 239 nM. Values of 0.32, 5.7, and 10.3 obtained from fura-2 *in situ* calibration were used for Rmin, Rmax, and β, respectively.

### Electrophysiological Recordings

STOCs were recorded in the whole cell configuration of the patch clamp technique using solutions and protocol previously described [48]. The pipette solution (in mmol/L): 80 Kglutamate, 5 NaCl, 40 KCl, 1 MgCl_2_, 3 MgATP, 0.1 NaGTP, 0.05 KEGTA, 20 HEPES, pH 7.2 with KOH. CASMCs were superfused with PSS and clamped at −50 mV or stepped from −50 to −10 mV in 10 mV increments. Membrane capacitance (Cm) was determined from the current amplitude elicited in response to a hyperpolarizing voltage pulse from a holding potential of −80 mV (duration, 10 ms; amplitude, 10 mV). Currents were filtered at 500 Hz and digitalized at 2 kHz (500 µs/point). STOC analysis was performed off line, using the event detection tool of Clampfit 9.2 (Axon Instruments, Inc); where both STOC time-to-rise and time-to-decay were measured at half peak of STOC in ms.

### Western Blots

Aorta homogenates were used because they provide more protein. They were prepared with homogenization buffer (in mmol/L: 300 sucrose, 20 HEPES, pH 7.2) plus protease inhibitors (1 µg/ml aprotinin, 500 µmol/L benzamidine, 12 µmol/L leupeptin, 100 µmol/L PMSF). Aortas were homogenized on ice with a glass homogenizer and spun at 2,000 *g* for 15 min at 4°C. Supernatants were fractionated on 15% SDS-PAGE gels, transferred onto nitrocellulose membranes (1 h at 100 V, Hybond-ECL, GE Healthcare Bio-Sciences Corp, NJ, USA) and probed with anti-BK α antibody (1∶1000; Alomone Labs, Jerusalem Israel), anti BK β1 antibody (1∶500; Alomone Labs, Jerusalem Israel), anti RyR (1∶5000; Thermo Scientific) and anti-actin antibody (1∶8000, Sigma-Aldrich) in phosphate-buffered saline solution containing Tween-20 (PBS-T; in mmol/L: 3 KH_2_PO_4_, 10 Na_2_HPO_4_, 150 NaCl, 0.1% Tween-20, pH 7.2–7.4). Membranes were incubated 1 h with secondary peroxidase-conjugated goat anti-rabbit (1∶15000). Signal was detected by chemiluminiscence. Densitometry was done with Kodak ID Software (v. 3.635, Molecular Imaging Systems). Actin signals were detected in the same blots. Protein concentration was assessed by the Bradford method.

### STOCs and Ca^2+^ Spark Analyses

Data are expressed as the mean ± SE. Statistical significance was assessed using SigmaStat 3.0 software by Student’s t-tests, one way ANOVA test or by nonparametric Mann-Whitney Rank-sum test for cases in which data failed the Shapiro-Wilk normality test. Values of *P*<0.05 were considered statistically significant. The probability density function analysis
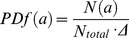
, where N(a) is the histogram distribution of Ca^2+^ spark parameter a, *N_total_* is the total number of Ca^2+^ sparks, and Δ is the bin width. Bimodal Gaussian distributions were done as previously reported [35].

## Supporting Information

Figure S1 A, Average of sites where Ca^2+^ sparks were recorded within the same cell during the recording period (9.6 seconds). Firing sites were counted as the sites where we recorded at least one Ca^2+^ spark. B, Ca^2+^ spark frequency within each firing site reported as number of events recorded within each site/s. C, Probability in each cell to present sites that fire repetitively. D, Maximum number of Ca^2+^sparks recorded within the same site. N = 43 for control CASMCs (*white bars*) and n = 41 for *db/db* cells (*gray bars*).
(DOCX)
