# Supplementary figures and images for: Abnormal Ca2+ Spark/STOC Coupling in Cerebral Artery Smooth Muscle Cells of Obese Type 2 Diabetic Mice
Source: PLoS One. 2013 Jan 3;8(1):e53321. doi: 10.1371/journal.pone.0053321 (PMC3536748; doi:10.1371/journal.pone.0053321)

**Figure S1**

**
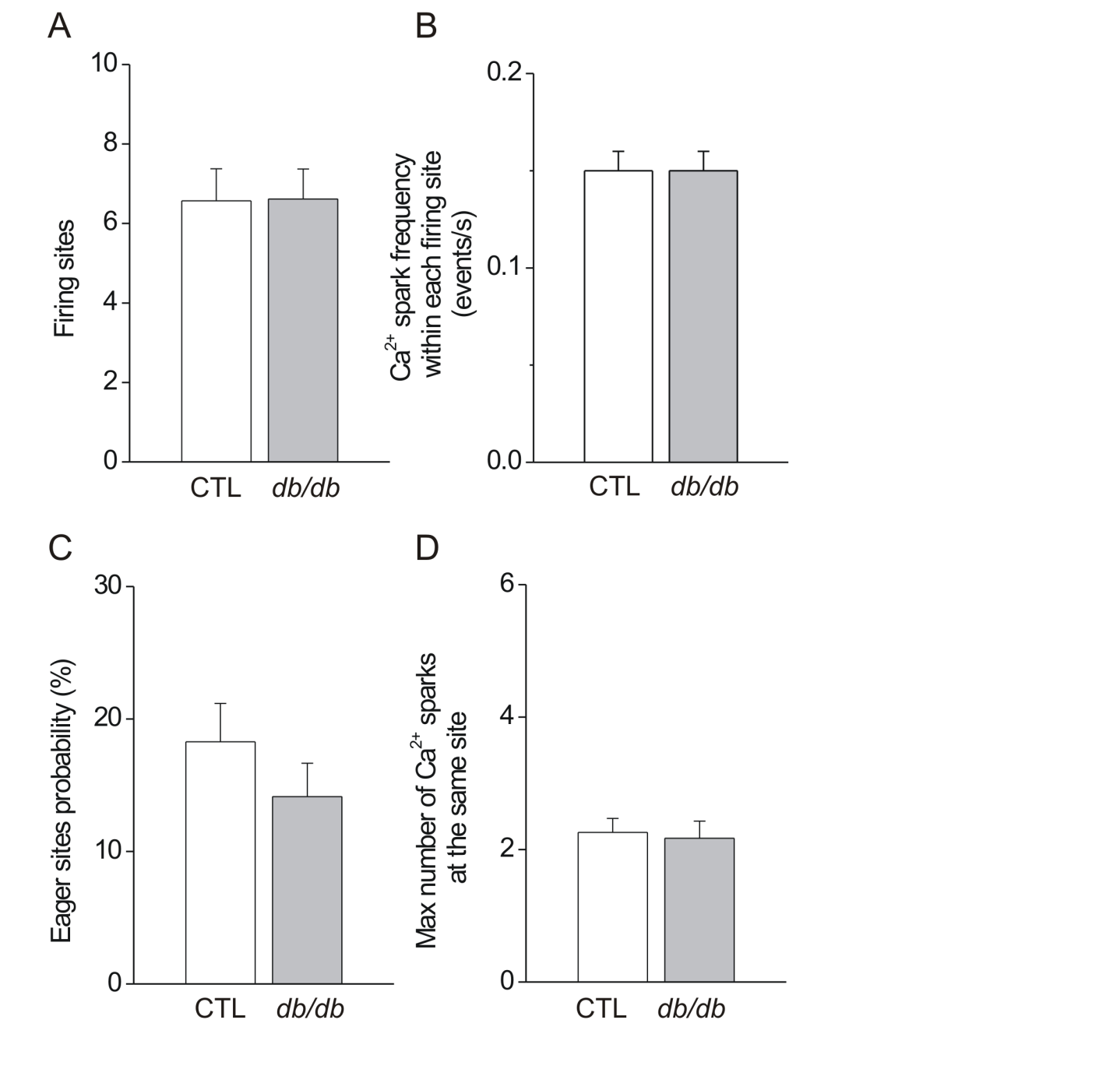
**

Supplement: Figure S1 — A, Average of sites where Ca2+ sparks were recorded within the same cell during the recording period (9.6 seconds). Firing sites were counted as the sites where we recorded at least one Ca2+ spark. B, Ca2+ spark frequency within each firing site reported as number of events recorded within each site/s. C, Probability in each cell to present sites that fire repetitively. D, Maximum number of Ca2+sparks recorded within the same site. N = 43 for control CASMCs (white bars) and n = 41 for db/db cells (gray bars). (DOCX) [file pone.0053321.s001.docx]
